# A 24-Month Follow-Up Study of the Effect of Intra-Articular Injection of Autologous Microfragmented Fat Tissue on Proteoglycan Synthesis in Patients with Knee Osteoarthritis

**DOI:** 10.3390/genes10121051

**Published:** 2019-12-17

**Authors:** Igor Borić, Damir Hudetz, Eduard Rod, Željko Jeleč, Trpimir Vrdoljak, Andrea Skelin, Ozren Polašek, Mihovil Plečko, Irena Trbojević-Akmačić, Gordan Lauc, Dragan Primorac

**Affiliations:** 1 St. Catherine Specialty Hospital, 49210 Zabok/10000 Zagreb, Croatia; damir.hudetz@svkatarina.hr (D.H.); eduard.rod@svkatarina.hr (E.R.); zeljko.jelec@svkatarina.hr (Ž.J.); andrea.skelin@svkatarina.hr (A.S.);; 2 School of Medicine, University of Split, 21000 Split, Croatia; 3 Medical School, University of Rijeka, 51000 Rijeka, Croatia; 4 School of Medicine, University of Mostar, 88000 Mostar, Bosnia and Herzegovina; 5 Clinical Hospital “Sveti Duh”, 10000 Zagreb, Croatia; trpimir.vrdoljak@gmail.com; 6 School of Medicine, JJ Strossmayer University of Osijek, 31000 Osijek, Croatia; 7 Department of Nursing, University North, 48 000 Varaždin, Croatia; 8 Genos Glycoscience Research Laboratory, 10000 Zagreb, Croatia; iakmacic@genos.hr (I.T.-A.); glauc@genos.hr (G.L.); 9 Faculty of Pharmacy and Biochemistry, University of Zagreb, 10000 Zagreb, Croatia; 10 Children’s Hospital Srebrnjak, 10000 Zagreb, Croatia; 11 Eberly College of Science, The Pennsylvania State University, University Park, State College, PA 16802, USA; 12 The Henry C. Lee College of Criminal Justice and Forensic Sciences, University of New Haven, West Haven, CT 06516, USA; 13 School of Medicine, Faculty of Dental Medicine and Health, University “Josip Juraj Strossmayer”, 31000 Osijek, Croatia

**Keywords:** mesenchymal stem cell, knee osteoarthritis, adipose tissue, regenerative medicine, dGEMRIC, glycosaminoglycans, cartilage

## Abstract

Osteoarthritis (OA) is a widely prevalent disease worldwide, and with an increasingly ageing society, it has become a challenge for the field of regenerative medicine. OA is a disease process involving multiple joint tissues, including those not visible on radiography, and is a complex disease process with multiple phenotypes that require evaluation by a multimodality imaging assessment. The purpose of this study was to evaluate the effect of micro-fragmented fat tissue intra-articular injection 24 months after application in two ways: Indirectly using functional magnetic resonance imaging (MRI) assessment analyzing the glycosaminoglycans (GAG) content in cartilage by means of delayed gadolinium (Gd)-enhanced magnetic resonance imaging of cartilage (dGEMRIC), as well as clinical outcome on observed level of GAG using standard orthopedic physical examination including VAS assessment. In our previous study assessing comprehensive results after 12 months, the dGEMRIC results have drawn attention. The present study explores the long-term effect of intra-articular injection of autologous microfragmented adipose tissue to host chondrocytes and cartilage proteoglycans in patients with knee OA. A prospective, non-randomized, interventional, single-center, open-label clinical trial was conducted from January 2016 to April 2018. A total of 17 patients were enrolled in the study, and 32 knees were assessed in a 12-month follow-up, but only 10 patients of them with 18 knees are included in a 24-month follow-up. The rest of the seven patients dropped out of the study 12 months after follow-up: three patients underwent knee arthroplasty, and the remaining four did not fulfil the basic criteria of 24 months involvement in the study. Surgical intervention (lipoaspiration), followed by tissue processing and intra-articular injection of the final microfragmented adipose tissue product into the affected knee(s), was performed in all patients. Patients were assessed for a visual analog scale (VAS), dGEMRIC at the baseline, three, six, 12 and 24 months after the treatment. A magnetic resonance sequence in dGEMRIC due to infiltration of the anionic, negatively-charged contrast gadopentetate dimeglumine (Gd-DTPA2) into the cartilage indicated that the contents of cartilage glycosaminoglycans significantly increased in specific areas of the treated knee joint. Our results suggest that this method of single intra-articular injection of autologous microfragmented adipose tissue improves GAG content on a significant scale, with over half of the measurements suggesting relevant improvement 24 months after intra-articular injection opposed to the expected GAG decrease over the natural course of the disease.

## 1. Introduction

Osteoarthritis (OA) is a widely prevalent disease worldwide, and with an increasingly ageing society, is has become a challenge for the field of regenerative medicine. In clinical practice and clinical trials, radiography remains the primary imaging modality for the evaluation of OA, however, limitations of radiography for the visualization of OA features, including insensitivity to early changes, non-specificity, absence of reproducibility in longitudinal studies and challenges regarding positioning, significantly limits the utility of radiography both clinically and in the research field. OA is a disease process involving multiple joint tissues including those not visible on radiography [1], and is a complex disease process with multiple phenotypes [2,3] that require evaluation by multimodality imaging assessment. Therefore, the use of more advanced imaging modalities such as magnetic resonance imaging (MRI) has become important in OA research [1,4]. For imaging of OA, MRI plays a major role in the research setting, with compositional MRI techniques becoming increasingly more important due to their capacity to assess ‘pre-morphologic’ biochemical compositional changes of articular and periarticular tissues [5]. Compositional MRI techniques can potentially supplement routine clinical MRI sequences to identify cartilage degeneration at an earlier stage when radiographs might be normal [6]. In this study, which is continuation of our previous study [7], we used delayed gadolinium (Gd)-enhanced magnetic resonance imaging of cartilage (dGEMRIC) to assess proteoglycan synthesis. dGEMRIC is a molecular imaging technique that has been used to study glycosaminoglycan (GAG) loss in the articular cartilage of patients with primary OA [7,8]. Additionally, it can be readily used on patients with a cruciate ligament injury, in order to evaluate associated cartilage lesions [8,9]. With dGEMRIC, T1-maps of hyaline cartilage are created following the intravenous (IV) administration of an anionic gadolinium-based contrast agent gadopentetate dimeglumine (Gd-DTPA2). Since the cartilage matrix is largely composed of GAG molecules with negatively-charged carboxyl and sulfate groups, it repels the negatively-charged contrast ions. As a result, the gadolinium concentrations are higher in the cartilage regions with low GAG concentrations, and the cartilage T1-relaxation time (T1Gd) is reduced [7,8]. The Gd-DTPA2 concentration per voxel is described by the means of the dGEMRIC index (T1Gd), which is calculated from the five different inversion times using a curve-fitting method. In areas with low GAG, the calculated T1Gd will be low, and vice versa. The resulting dGEMRIC index (the average T1Gd in a region of interest) is related to both the GAG concentration and the time between gadolinium administration and image acquisition [10,11]. 

Therefore, healthy cartilage that contains an abundance of GAGs will have low concentrations of Gd-DTPA2, whereas degraded cartilage will have high concentrations of the contrast agent in the areas where GAGs have been lost. T1-relaxation times are inversely proportional to the concentration of Gd-DTPA2, and thus provide a quantitative metric of cartilage integrity [12,13,14]. In this study, we hypothesized that there are changes in the composition of the articular cartilage extracellular matrix after administration of autologous and microfragmented fat tissue in patients with knee osteoarthritis that are detectable by the means of pre-structural cartilage assessment using MRI [7]. We enrolled patients with Kellgren Lawrence osteoarthritis stages III and IV, and followed the effects of the single-shot administration of microfragmented fat tissue with adipose-derived stem cells (ASCs) during 24 months using MRI. To the best of the authors’ knowledge, this is the first study to evaluate the effect of autologous and micro-fragmented adipose tissue injection on the articular cartilage of the knee joint facets using the dGEMRIC technique with a 24-month longitudinal assessment of GAG content within the cartilage.

## 2. Patients and Methods

### 2.1. Study Design

A prospective, non-randomized, interventional, single-center, open label clinical trial of a single intra-articular injection of autologous microfragmented adipose tissue containing Ad-MSCs in patients with primary knee osteoarthritis was conducted from January 2016 to April 2018 in the St. Catherine Specialty Hospital, Zabok/Zagreb, Croatia. The aim of this study was to evaluate the effect of micro-fragmented fat tissue intra-articular injection 24 months after application in two ways: Indirectly, using functional MRI assessment analyzing the GAG content in cartilage by means of Dgemric, as well as clinical outcome on observed level of GAG using standard orthopedic physical examination including VAS assessment. The study protocol was approved by the local Institutional Review Board (IRB) under authorization No: EP 001/2016. The study was registered in ISRCTN (ID: ISRCTN13337022). Patients with primary knee OA who satisfied the inclusion criteria (radiological Kellgren Lawrence grade III-IV; onset of symptoms of the index knee at six or more months ago; ability to follow the instructions of the study; aged 40–85) were enrolled in the study. The exclusion criteria were: age < 40 years or > 85 years; chondromatosis or villonodular synovitis of the knee; recent trauma (<3 months) of the symptomatic knee; infectious joint disease; malignancy; pregnancy; patients on anticoagulant therapy with prothrombin time (PT) (<0.70), or suffering from thrombocytopenia and/or coagulation disorder; hypersensitivity to local anesthetics. Participants were assessed using a detailed clinical history, a complete physical examination and a radiological assessment including plain X-rays (AP standing and LL knee projections), full-length weight-bearing (FLWB) X-ray in the standing position in order to measure limb alignment, MRI, dGEMRIC and biochemical laboratory test results. Additional assessment included the VAS pain scale. Patients were assessed at time points 0 months, 3 months, 6 months 12 months and 24 months after the intervention. A total of 17 patients were enrolled in the study, and 32 knees were assessed in a 12-month follow-up, but only 10 patients of them with 18 knees are included in a 24-month follow-up. The rest of the seven patients dropped out of the study after 12 months follow up: Three patients underwent to knee arthroplasty and the remaining four did not fulfil basic criteria and stay in the study for 24 months. Patients received detailed written and oral information about the study protocol, and were asked to sign an informed consent form. Once they entered the trial, patients were assigned a unique anonymous code, and data was collected in the data logbook. Baseline information collected in the registry included primary diagnoses, medical history and patient demographics. All procedures were standardized and implemented according to the standard operating procedure protocol (SOP). 

### 2.2. Transplantation and Processing of Microfragmented Adipose Tissue

The patients were referred to a day surgery unit with an average admission of 3 h. The surgical part of the procedure was set up in an operating theater. Patients were placed in a supine position; the abdominal skin was treated with antiseptic lotion Dermoguard^®^ (Antiseptica, Pulheim, Germany), rinsed with Aqua pro injection solution (HZTM, Zagreb, Croatia) and dried out and disinfected with Skin-Des^®^ solution (Antiseptica). The minimal invasive surgical procedure included an infiltration step, in which a total of 250 mL of saline solution prepared with a 40 mL of a 2% lidocaine solution (Lidokain^®^, Belupo, Koprivnica, Croatia) and 1 mL epinephrine hydrochloride (1 mg/mL) (Suprarenin^®^, Sanofi-Aventis, Berlin, Germany) was injected in the abdominal subcutaneous adipose tissue. In the aspiration step, a standard lipoaspiration technique was performed, and the harvested fat was introduced into the Lipogems^®^ ortho kit (Lipogems International SpA, Milan, Italy) according to the manufacturer’s instructions, as previously described [15]. The collected and processed final microfragmented adipose tissue product was transferred to several 10 mL syringes and injected intra-articularly (4–15 mL) into the index knee. Immunophenotyping of a stromal vascular fraction from microfragmented lipoaspirate was also done [16].

### 2.3. MR Imaging

MR imaging was performed on a 1.5 T magnet (Avanto; Siemens, Erlangen, Germany) using a dedicated knee coil (Siemens). Five turbo spin-echo inversion recovery sequences (inversion times of 1650 ms, 650 ms, 350 ms, 150 ms and 28 ms; repetition time (TR) = 1800 ms; time to echo (TE) = 19 ms; bandwidth = 326 Hz; field of view (FOV) = 160 mm; matrix = 384 × 384; voxel size = 0.4 × 0.4 × 3 mm; number of excitations (NEX) = 1) were then acquired for subsequent T1Gd mapping to compute the dGEMRIC indices of femoral, tibial and patellar articular cartilage. The severity of early OA in the study cohort was determined according to the MRI scan by an experienced musculoskeletal radiologist using the scoring system introduced by the International Cartilage Research Society (ICRS) based on a modified Outerbridge system divided into five stages according to cartilage lesion size and depth, as well as the appearance of the surrounding subchondral bone: Grade 0: normal cartilage; Grade 1: signal intensity alterations with an intact surface of the articular cartilage compared with the surrounding normal cartilage; Grade 2: partial-thickness defect of the cartilage with fissures on the surface that do not reach the subchondral bone or exceed 1.5 cm in diameter; Grade 3: fissuring of the cartilage to the level of the subchondral bone in an area with a diameter more than 1.5 cm; Grade 4: exposed subchondral bone. The status of the cartilage was analyzed on seven different articular facets: medial and lateral femoral condyle, femoral trochlea, medial and lateral tibial condyle and both patellar facets. In addition, the thickness of the articular cartilage was measured in the same place before the intra-articular application of stem cells, and in every subsequent MRI examination.

### 2.4. dGEMRIC Protocol

Each subject received gadolinium diethylene triamine penta-acetic acid (Dotarem; Guerbet, Roissy CgG Cedex, Villepinte, France), 0.2 mmol/kg, administered by slow IV infusion through a catheter placed in the antecubital vein with the patient in the supine position in order to avoid thrombophlebitis at the injection site [17]. The relaxivity of the administered MRI contrast agent was the same for all patients, because the MRI contrast agent was always applied under the same conditions: contrast agent temperature, magnetic field strength and contrast agent concentration. The contrast agent injection time was less than 5 min. The subject then exercised by walking up and down the stairs for approximately 10 min, starting 5 min after injection, to promote the delivery of the contrast agent to the joint. Post-contrast imaging of the cartilage was performed 120 min after contrast administration. The dGEMRIC images were analyzed by an experienced musculoskeletal radiologist using the syngoMaplt software (Siemens). 

The dGEMRIC index was analyzed on seven different articular facets: the medial and lateral femoral condyle, femoral trochlea, medial and lateral tibial condyle and both patellar facets, before the intra-articular application of stem cells and in any subsequent MRI examination at three, nine, twelve and 24 months after the intra-articular application of stem cells. Regions of interest (ROIs) in which an average T1 index was calculated, were manually drawn to always cover the same central (weight-bearing) part of each articular facet. Articular facets without a cartilage cover, where it was not possible to measure the dGEMRIC index, were labeled as “0”, and articular facets on which, for some reason, the dGEMRIC index was not measured, were labeled as “-”. The highest differences between various measurements within our study at the same ROI were below 6% (range 0.1% to 5.4%) and corresponded with data reported in other studies [17].

### 2.5. Statistical Analysis

We used descriptive and inferential methods to analyze the data. Means and standard deviations were used to estimate central tendency and variability. Numerical data were analyzed using the t-test, and the chi-square test was used for categorical variables. We used the pairwise t-test for paired and subsequent measurements of the same patient or a knee. Within-individual variation was defined on the basis of the dGEMRIC error rates, which were based on previously published papers, and this indicated that the mean difference per region of interest between the two T1Gd measurements ranged from 3.7% to 6.8% [14,16,18]. Based on this, we defined the arbitrary change of 15% in subsequent measurements as clinically relevant, and considered this as the liminal value (on the basis of two standard deviations from the estimated error rate). All analyses were performed in R (http://www.r-project.org/), with significance set at p < 0.05.

### 2.6. VAS protocol

Numeric rating scale (RS), a segmented numeric version of the visual analog scale (VAS) in which a respondent selects a whole number (0–10 integers) that best reflects the intensity of their pain was used [19].

## 3. Results

### 3.1. Patient Characteristics

Seventeen patients that matched the inclusion criteria were consecutively allocated for the study, and received an intra-articular injection of microfragmented fat tissue with Ad-MSCs, but only 10 patients of them stayed in the study for the 24-month follow up. Generally, all the patients enrolled in the study showed similar baseline characteristics of age (mean 69 ± 12), height, weight, body mass index (BMI) and radiographic Kellgren Lawrence grade of osteoarthritis (grade III or IV). Sex distribution was: seven male and three female patients. The pattern, distribution and severity of cartilage tissue deterioration as assessed by MRI, and dGEMRIC varied substantially at the baseline.

The FLWB X-ray in the standing position revealed mechanical overload patterns of the worn knee compartments with a complete lack of the cartilage layer. Out of 18 knees, we observed 15 to show Varus deformities, with an average malalignment of 8.6; three valgus knees with an average two of deformities, and one perfectly aligned knee.

### 3.2. Delayed Gadolinium-Enhanced MRI of Cartilage (dGEMRIC)

The dGEMRIC index was measured on seven different articular facets for each knee in the study, and the results are presented in a separate table for each patient (Figures S1–S10). The dGEMRIC index was presented in absolute values, and the change of the index value through the study period is shown in percentages in relation to the baseline dGEMRIC index value. 

We estimated a percentage change of the dGEMRIC results and estimated the number of clinically relevant improvements vs. deteriorations in each patient (Figures S1–S10), where a 15% change was considered a relevant change (based on the error rates acquired from the available literature and our studies) [14].

A total of ten patients were followed up for 24 months. In total, there were 76 measurements of the dGEMRIC index with an average change of +2.7 ± 17.2 (with very large SD due to the fact that positive and negative values were used in this analysis). Two years after intra-articular injection of autologous, microfragmented adipose tissue, 19 measurements were showing changes with a greater magnitude than 15% (in our previous study, we had defined the clinically relevant threshold as change of at least 15%; this cut-off value was based on the inherent measurement error of 7%, which was doubled in order to define the cut-off value). Out of these relevant changes, there were 12 improvements when compared to the beginning (64.2%), while the remaining seven were showing relevant decrease (36.8%); the difference in frequency of change was not significant (P = 0.251; under the equal expected frequency assumption). In 57 other measurements, the dGEMRIC index was showing the change that was of lower magnitude than 15%. Furthermore, when analyzing effects of intra-articular injection of autologous, microfragmented adipose tissue two years after application, we noted that the extent of magnitude between positive and negative cases was uneven; average decrease was −16.2 ± 1.5%, while the average increase was 32.3 ± 21.9%, what was marginally significant when absolute values were used (P = 0.028). Improvements were most commonly observed in the area of the lateral tibial condyle (four increased, one decreased) and lateral femoral condyle (four increased, three decreased). We also observed the highest decrease of the values of the medial femoral and medial tibial facets, in patients with Varus knee deformities.

Pooling all seven locations into an individual-level percent change indicated large inter-personal differences, ranging from −14.7% to +26.0% average change. Due to these differences, we had reported all observed patients individually (Figures S1–S10). Lastly, we also performed a comparison of the VAS between t0 and t24; resting VAS had decreased from 4.45 ± 2.42 to 0.55 ± 1.04 (p < 0.001), while activity VAS had decreased from 7.73 ± 1.35 to 3.40 ± 1.65 (p< 0.001).

By analyzing the dGEMRIC index values changes 24 months after intra-articular injection of microfragmented fat tissue with severity of cartilage lesions, we did not find a significant deviation of dGEMRIC index changes between ICRS grade II and grade III lesions. In cartilage with ICRS grade 4 lesions, there were no index value changes. 

## 4. Discussion

The evaluation of a treatment response in patients with primary knee OA is a great challenge. We use a single intra-articular injection of autologous microfragmented adipose tissue; the novelty of this approach is the use of tissue instead of cells, avoiding: [1] regulatory constraints and [2] using a tissue with an intact niche. The evaluation used MRI because of the ability of magnetic resonance (MR) to image structures within the knee and to visualize cartilage morphology and composition, which is a critical role in understanding the repair mechanisms. The patients group enrolled in the study had knee OA grade III and IV, meaning that we were able to document diverse statuses of the cartilage layer within the joint on MRI scans. We were able to follow the effects of the treatment on full-thickness cartilage layers, as well as on completely destroyed cartilage layers with exposed sclerotic subchondral bone. This approach has shown to be very descriptive for evaluating the effects of the treatment. To the best of the authors’ knowledge, this is the first study to evaluate the effect of autologous and micro-fragmented adipose tissue injection on articular cartilage of the knee joint facets using the dGEMRIC technique with a 24-month longitudinal assessment of GAG content within the cartilage. A total of 17 patients were enrolled in the study, and 32 knees were assessed in 12-month follow-up [7], but only 10 patients of them with 18 knees were included in a 24-month follow-up. The rest of the seven patients dropped out of the study after 12 months follow-up: three patients underwent to knee arthroplasty, and the remaining four patients were lost to follow-up after 12 months (unrelated reasons with the study: illness, relocation from the country…).

The highest differences between various measurements at the same ROI in our study were below 7% (range from 0.1% to 5.4%), which corresponds to published data [17]. There was no influence of the different relaxivity of the administered MRI contrast agent on the dGEMRIC index value because the same MRI contrast agent was always applied, and administration was always performed under the same conditions: contrast agent temperature, magnetic field strength and contrast agent concentration [20]. 

Comparison of dGEMRIC index value after 24 months with baseline measured at the specific region of interest in seven different joint facets show that the average change was +2.7 ± 17.2 (with very large SD due to the fact that positive and negative values were used in this analysis). As much as 19 measurements were showing change that was of greater magnitude than 15% (in our previous study, we had defined the clinically relevant threshold as change of at least 15%; this cut-off value was based on the inherent measurement error of 7%, which was doubled in order to define the cut-off value). Out of these relevant changes, there were 12 improvements when compared to the beginning (64.2%), while the remaining seven were showing relevant decrease (36.8%). We found high correlation of dGEMRIC index improvements with resting and activity VAS decrease. An increase in index value was for the most articular facets ICRS grade II and III chondromalacia after autologous and microfragmented adipose tissue injection. As expected, in cartilage lesions International Cartilage Repair Society (ICRS) grade IV, there were no changes in dGEMRIC value through the study period.

Interestingly, when we compared results from our previously published article [7] assessing the one year effect after intra-articular injection of autologous microfragmented adipose tissue, where out of 331 dGEMRIC measurements, 175 measurements (82.6%) were showing changes with a greater magnitude than 15%, while the remaining 37 dGEMRIC measurements (17.5%) were showing relevant decrease, it become clear that the intra-articular injection of autologous microfragmented adipose tissue plays a significant role in GAGs production even two years after the treatment.

VAS results obtained from the patients at index time and after 24 months have shown substantial significant decrease over the time. The observed effect of treatment measured with VAS values has been more pronounced than the GAG content changes captured with dGEMRIC. One could speculate that the effect of treatment might be diminished with longer than 24 months’ follow-up, and that the changes in dGEMRIC values are detectable prior to the changes in the level of pain. Reduction of the joint effusion, decrease in pain sensation on the VAS scale and increase in range of movement enabled more intensive exercising and rehabilitation [21]. This might have had an impact on improving GAG concentration. In a study of Choi at al., individuals exercising on a regular basis were shown to have higher dGEMRIC indices (i.e., higher GAG concentrations) than sedentary individuals, correlating with the level of physical activity [22]. In another study, a change in dGEMRIC index was shown four months after a meniscal tear, corresponding to the amount of exercise [23]. This study provided an insight into the hypothesis that mechanical stimulation could also change cartilage biochemistry.

We also observed the highest decrease of the values of medial femoral and medial tibial facets, in patients with Varus knee deformities, due to increased weight load. 

The medial femorotibial compartment has been shown to have a generally lower dGEMRIC index compared with the lateral femorotibial compartment of the knee, consistent with previous biochemical studies, as well as possibly reflecting a response to different mechanical stresses, according to the location within the joint [21].

Varus deformity of 5 or more degrees is an exclusion criterion for some of the published papers [24,25]. Some studies showed an effect of microfragmented adipose tissue in combination with other procedures like high tibial osteotomy [26] or the effect of mesenchymal stem cells derived from the infrapatellar fat pad in combination with PRP [25,27].

The 24-months follow up results comparing with 12 months results suggested that after 12 months index value, indirectly representing GAG content, decreased in most of the patients, but was still higher in more than half of patients in comparison to the baseline values.

Our results suggest that this method of single intra-articular injection of autologous microfragmented adipose tissue improves GAG content on a significant scale, with over half of the measurements suggesting relevant improvement 24 months after intra-articular injection in opposed to the expected GAG decrease over the natural course of the disease.

## Supplementary Materials

The following are available online at https://www.mdpi.com/2073-4425/10/12/1051/s1, Figure S1: The scheme of the dGEMRIC index with different joint facets throughout the study period at T0, T12 and T24 combined with VAS scale ratings in T0, T12 and T24 for patient dG01; Figure S2: The scheme of the dGEMRIC index with different joint facets throughout the study period at T0,T12 and T24 combined with VAS scale ratings in T0, T12 and T24 for patient dG02; Figure S3: The scheme of the dGEMRIC index with different joint facets throughout the study period at T0,T12 and T24 combined with VAS scale ratings in T0, T12 and T24 for patient dG05; Figure S4: The scheme of the dGEMRIC index with different joint facets throughout the study period at T0,T12 and T24 combined with VAS scale ratings in T0, T12 and T24 for patient dG07; Figure S5: The scheme of the dGEMRIC index with different joint facets throughout the study period at T0,T12 and T24 combined with VAS scale ratings in T0, T12 and T24 for patient dG08; Figure S6: The scheme of the dGEMRIC index with different joint facets throughout the study period at T0,T12 and T24 combined with VAS scale ratings in T0, T12 and T24 for patient dG09; Figure S7: The scheme of the dGEMRIC index with different joint facets throughout the study period at T0,T12 and T24 combined with VAS scale ratings in T0, T12 and T24 for patient dG15; Figure S8: The scheme of the dGEMRIC index with different joint facets throughout the study period at T0,T12 and T24 combined with VAS scale ratings in T0, T12 and T24 for patient dG16; Figure S9: The scheme of the dGEMRIC index with different joint facets throughout the study period at T0,T12 and T24 combined with VAS scale ratings in T0, T12 and T24 for patient dG18; Figure S10: The scheme of the dGEMRIC index with different joint facets throughout the study period at T0,T12 and T24 combined with VAS scale ratings in T0, T12 and T24 for patient dG17.
